# Nitrate of Silver and Ipecacuanha in Diarrhœa and Dysentery

**Published:** 1842-05

**Authors:** 


*Nitrate of Silver and Ipecacuanha in Diarrhœa and Dysentery.*—Prof. Trousseau, of the Necker Hospital, has employed this combination in both the acute and chronic forms of these two diseases, and with great success. In a late number of the *Journal des Connaissances Medico-Chirurgicales* we find a report of several of the cases. In cases of dysentery an emetic of ipecacuanha was first given, and then an enema containing 25 centigrammes (5 *grains*) of nitrate of silver in 20 ounces of water. The dysentery was often checked by a single enema, but the treatment was generally continued for four or five days. It was equally useful, according to Prof. Trousseau, whether the disease occupied the colon or the ilium, only in the latter case, instead of enemata the medicine was given in pill—the dose 2½ centigrammes (½ *grain*) to an adult. To a child with chronic diarrhœa, 1–5th grain was given by the mouth, or a grain in enema.—*N. Y. Med. Gaz.*

